# Fenretinide regulates macrophage polarization to protect against experimental colitis induced by dextran sulfate sodium

**DOI:** 10.1080/21655979.2020.1859259

**Published:** 2020-12-31

**Authors:** Yong Cao, Yan Lin, Yan Sun, Weiyu Liu, Yichuan Shao, Changqing Zheng

**Affiliations:** a Department of Gastroenterology, Shengjing Hospital of China Medical University, Shenyang, People’s Republic of China; b Department of Gastroenterology, The People’s Hospital of Liaoning Province, Shenyang, People’s Republic of China; c School of Information Engineering, Shenyang University, Shenyang, People’s Republic of China

**Keywords:** Fenretinide, ulcerative colitis, macrophage polarization, PPAR-γ, RAW264.7 cells

## Abstract

Fenretinide (4-HPR), a synthetic retinoid, has attracted attention for its anti-inflammation activity. However, few studies have evaluated the effects of 4-HPR on ulcerative colitis (UC). The present study was performed to investigate the therapeutic effects of 4-HPR on UC, and to explore the mechanisms mainly focused on macrophage polarization involved in this progress. Intraperitoneally administered 4-HPR particularly at dose of 100 mg/kg obviously alleviated UC symptoms and restrained the mRNA expression of colonic IL-1β, IL-6, and TNF-α in dextran sulfate sodium (DSS)-induced mice. Further analysis showed that 4-HPR decreased the mRNA expression of M1 macrophage markers IL-12 and iNOS, while increased M2 macrophage markers Ym1, Arg1 and MRC1 in colonic tissue of mice received DSS. Consistently, an *in vitro* study revealed that 4-HPR decreased inflammatory response and M1 polarization, while enhanced M2 polarization in LPS-induced RAW264.7 cells. Interestingly, 4-HPR remarkably activated PPAR-γ which was an important regulator of macrophage polarization both in colonic tissue of UC mice and in LPS-induced RAW264.7 cells. Furthermore, these effects of 4-HPR *in vivo* and *in vitro* including anti-inflammation and modulation of macrophage polarization were partially abolished by treatment with PPAR-γ antagonist GW9662, indicating that 4-HPR activated PPAR-γ to exert its activities. Taken together, this study demonstrated that 4-HPR might be a potent anti-UC agent that works by regulating macrophage polarization via PPARγ.

## GRAPHICAL ABSTRACT

**Figure:**
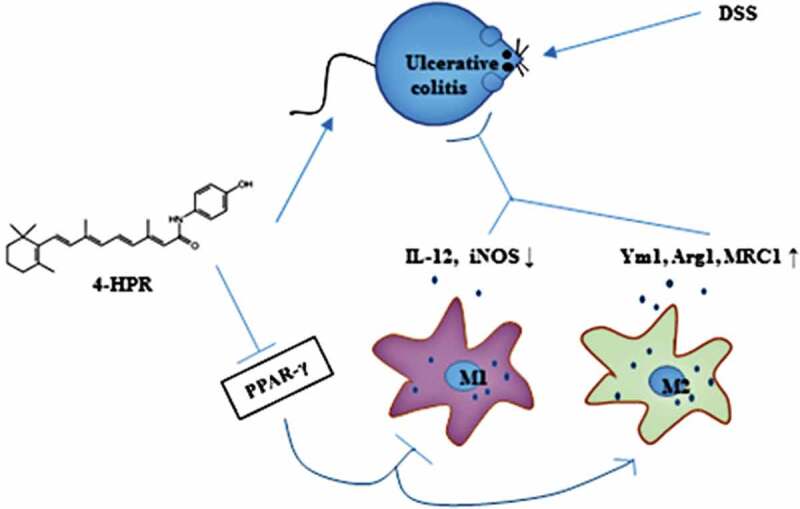

## Introduction

Crohn’s disease and ulcerative colitis (UC) are collectively termed inflammatory bowel disorders. UC is a recurrent intestinal inflammation disease, characterized by epithelial cell destruction, mucosa ulceration, and inflammatory cell infiltration in intestine and/or colon [1]. UC patients present with clinical symptoms such as abdominal pain, diarrhea, and bloody purulent stool. The initial treatment strategies of UC usually follow the classic step-up method. 5-aminosalicylic acid, steroids, and immunosuppressant are usually applied. Unfortunately, these treatment strategies only have limited efficacy and cause severe side effects [2]. Common adverse events of 5-aminosalicylic acid application included flatulence, abdominal pain, nausea, diarrhea, and headache [3]. Immunesuppressants such as azathioprine (AZA) and mercaptopurine (MP) have their own potential risks including, but not limited to, myelosuppression and liver toxicity [2]. Furthermore, acute episode of severe colitis occurs in 15% of UC patients, and 30% of these critically ill patients require surgical therapy, which seriously affects the patients’ quality of life [4–6]. Thus, it is urgent to find novel agents for UC treatment.

Macrophages are innate immune cells that are strategically positioned throughout the body tissues, including intestinal tissue. Previous studies have reported that colonic macrophages orchestrate inflammatory processes associated with UC [7,8]. The activation state of macrophages depends on the changes of tissue microenvironment. Activated macrophages with pro-inflammatory properties are known as M1-type macrophages. In addition, macrophages can also become alternatively activated (M2-type) macrophages, mediating anti-inflammatory responses. In DSS-induced colitis, transferring from the M1 to M2 macrophages was protective, M1 macrophages were increased while the population of M2 macrophages was decreased [9,10]. M1 macrophages produce a large number of inflammatory mediators and nitric oxide causing intestinal tissue damage. In contrast, M2 macrophages are considered as intestinal tissue repair coordinators. Thus, regulating macrophages polarization might become an emerging target for UC treatment.

Fenretinide (4-HPR) is a synthetic analog of retinoid (vitamin A) with potential chemopreventive activities. People found that 4-HPR exhibited better tissue distribution with lower toxicity than its natural congeners and other synthetics [11]. Evidence suggested that 4-HPR inhibited the production of macrophage inflammatory mediators via PPARγ pathway, and attenuated inflammation in allergic asthma [12,13]. In addition, 4-HPR could regulate macrophages polarization in colon cancer [14]. Nevertheless, few studies have evaluated the effects of 4-HPR on UC.

In the present study, we sought to examine whether 4-HPR has protective properties against UC and to explore the mechanisms focused on the macrophage polarization in this process.

## Materials and methods

### Animals

Male C57BL/6 mice were obtained from Liaoning Changsheng Biotechnology Co, Ltd. (Benxi, China). Mice were housed at 25 ± 1°C with *ad libitum* access to food and water, and under a fixed 12-h light-dark cycle. All animal experiments were performed in accordance with the guide for the Care and Use of Laboratory Animals published by the National Institutes of Health and approved by the Ethics Committee of Shengjing Hospital of China Medical University (2018PS71K).

### DSS-induced colitis establishment and drug treatment

After a week of environment adaption, the mice were divided into five groups: Control group, UC group, UC with 4-HPR-L (50 mg/kg)-treated group, UC with 4-HPR-H (100 mg/kg)-treated group, and UC with 4-HPR-H (100 mg/kg) and PPARγ antagonist GW9662 (1 mg/kg)-treated group. The UC was induced by giving 3% DSS in the drinking water for 7 continuous days. 4-HPR and GW9662 were intraperitoneally injected every day. Twenty-four hours after the last administration, all mice were killed by intraperitoneal injection with pentobarbital sodium (200 mg/kg).

### Disease activity index (DAI)

The UC was evaluated based on colon length and DAI. DAI, as the mean scores of body weight loss, stool consistency and stool blood, was scored day 1 to day 7 during the experimental period. The scoring criteria were based on the previous report [15].

### Histopathological assessment

The colon length was recorded after sacrifice of the mice. Then, colonic tissues were fixed and then dehydrated for paraffin embedding. Sections were next cut into 5-μm serial sections and then stained with hematoxylin and eosin (H&E) according to standard protocols. Pathological photographs were obtained with an Olympus BX53 microscope (Olympus, Tokyo, Japan) at 200× magnification.

### Detection of myeloperoxidase (MPO) and prostaglandin E2 (PGE_2_)

Colon tissues were homogenized for the detection of MPO and PGE_2_. MPO activity was measured using a commercial kit (Nanjing JianCheng, Nanjing, China). The detection of PGE_2_ was performed using a PGE_2_ ELISA assay kit (USCN, Wuhan, China).

### Immunohistochemistry assay

Immunohistochemistry assay was performed for the detection of COX-2 in the colonic tissues. Colonic tissues were fixed with formaldehyde, dehydrated and embedded in paraffin. Then, the sections were cut into 5-μm, deparaffinized, hydrated, and boiled in antigen retrieval buffer. After elimination of endogenous peroxidase activity with 3% hydrogen peroxide, sections were blocked with goat serum, then incubated with antibody against anti-COX2 (1:200 dilution, abcam, Cambridge, UK) overnight at 4°C. Hereafter, sections were incubated with the corresponding secondary antibody (1:200 dilution, thermoFisher, Waltham, MA, USA) at 37 °C for 1 h, and visualized with diaminobenzidine (Solarbio, Beijing, China). Hematoxylin (Solarbio) was used for nuclei counterstaining. Photographs were obtained with an Olympus BX53 microscope (Olympus) at 400× magnification.

### Cell culture

Murine RAW264.7 cells (Procell, Wuhan, China) were cultured in DMEM complete medium (Gibco, Shanghai, China) at an atmosphere of 5% CO_2_ at 37°C. Cells were stimulated with LPS (1 μg/ml) for 24 h, then incubated with various concentrations of 4-HPR (L, 1 μM; H, 10 μM) for 24 h. In some experiments, the PPARγ antagonist GW9662 (5 μM) was added to the cells 1 h before treatment with LPS and 4-HPR-H (10 μM).

To determine the polarized state of RAW264.7 cells with different administrations, the level of M1 type surface marker CD86 (Biolegend Company, Beijing, China) and M2 type surface marker CD206 (Biolegend Company) was tested by flow cytometry according to standard protocols.

### Quantitative real-time PCR

Total RNA from colonic tissues or cells was isolated with a Total RNA Rapid Extraction Kit (BioTeke, Beijing, China), and reverse-transcribed to produce cDNA using Super M-MLV reverse-transcriptase (Takara, Beijing, China). Next, SYBR-based real-time PCR was performed to quantify IL-6, IL-1β, TNF-α, IL-12, iNOS, Ym1, Arg1, MRC1 and β-actin (housekeeping gene control) using ExicyclerTM 96 fluorescence quantifier. Relative expression of these genes was calculated using the 2^−ΔΔCT^ method and presented as fold-changes. Primer sequences are listed in Table 1.

**Table 1.** Primers for RT- PCR

Gene	sequence
IL-1β Forward	CTCAACTGTGAAATGCCACC
IL-1β Reverse	GAGTGATACTGCCTGCCTGA
IL-6 Forward	TGTATGAACAACGATGATGCAC
IL-6 Reverse	CTGGCTTTGTCTTTCTTGTT
TNF-α Forward	CGTCGTAGCAAACCACCAA
TNF-α Reverse	GGGCAGCCTTGTCCCTTGA
IL-12 Forward	GTTCCAACAGCCTCACCCTC
IL-12 Reverse	TTCGGGACTGGCTAAGACAC
iNOS Forward	CACCACCCTCCTCGTTC
iNOS Reverse	CAATCCACAACTCGCTCC
Ym1 Forward	ATGAGTGGGTTGGTTATG
Ym1 Reverse	AGTAGATGTCAGAGGGAAA
Arg1 Forward	GGAAGACAGCAGAGGAGGTG
Arg1 Reverse	TCAGTCCCTGGCTTATGGTT
MRC1 Forward	AGTGATGGTTCTCCCGTTTC
MRC1 Reverse	TGGGCTCAGGTAGTAGTGTTTT

### Western blot

Nuclear proteins were isolated with a nuclear extraction kit (beyotime) according to the instruction of the manufacturers. After quantification by a BCA protein assay kit (beyotime), proteins were separated by SDS-PAGE and transferred onto PVDF membranes. After blocking at room temperature for 1 h in nonfat 5% dry milk, the members were incubated in the diluted primary antibodies anti-PPARγ (1: 500 dilution, Abcam), anti-Histone H3 (1: 2000 dilution, Abgent, San Diego, CA, USA) at 4°C overnight. Hereafter, the members were incubated with the diluted secondary antibody (1: 5000, beyotime). Histone-H3 were used as the nucleic internal control. Blots were visualized using the Enhanced chemiluminescence (beyotime). Quantification of bands intensities were acquired with the Gel-Pro-Analyzer software.

### Statistical analysis

All data are reported as mean ± SD. A comparison of the results was performed with by Kruskal-Wallis test followed by Dunn’s multiple comparison test, one-way or two-way analysis of variance by Bonferroni test (Graphpad 8.0, Inc, San Diego, CA, USA). Statistical significance was defined as p < 0.05.

## Results

### 4-HPR alleviated DSS-induced UC symptoms in mice

**Figure 1.**
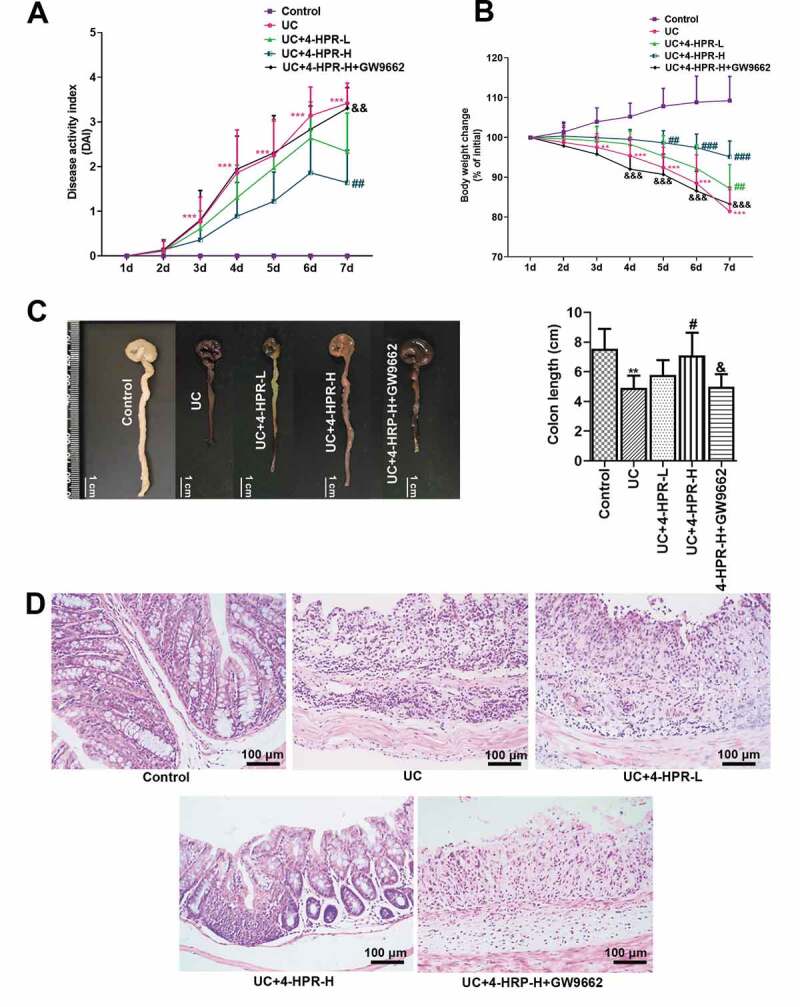
4-HPR alleviated DSS-induced UC in mice. (a) DAI during experiment period. (b) Body weight change during experiment period. (c) Representative photos of colons and colon length (cm). (d) Representative images of colon tissues with H&E staining (× 200). Data are shown as mean ± SD. **p < 0.01, ***p < 0.001 vs. Control group; ^#^p < 0.05, ^##^p < 0.01, ^###^p < 0.001 vs. UC group; ^&^p < 0.05, ^&&^p < 0.01, ^&&&^p < 0.001 vs. UC+4-HPR-H group

UC model was established in male C57BL/6 mice by administration 3% DSS. The results showed that DAI and body weight loss in the UC group was obviously increased than that in the Control group, reflecting the development of clinical symptoms of UC. However, 4-HPR at a dosage of 100 mg/kg significantly reduced DAI and body weight loss in UC mice (Figure 1(a,b)). After the mice were sacrificed, we measured colon length. There was a significant shortening of the colon length in DSS-induced mice. While the change of colon length was inhibited by 4-HPR administration (Figure 1(c)). Interestingly, GW9662 significantly diminished the inhibitory effects of 4-HPR-H on the clinical symptoms of UC and colon shortening in DSS-administrated mice (Figure 1(a-c)). Moreover, H&E staining analysis showed thickening of the colonic wall, loss of goblet cells and infiltration of the inflammatory cells, while all those traits in slices were significantly alleviated by 4-HPR (especially 4-HPR-H) administration. GW9662 dramatically weakened the action of 4-HPR-H on histopathological changes in colons (Figure 1(d)).

In addition, we confirmed the sole effect of 4-HPR (100 mg/kg) on mice. As shown in supplemental Figure 1(a,b), mice body weight and DAI did not show any marked changes in the 4-HPR (100 mg/kg) group, which was similar to the Control group. Similarly, mice in the high dosage reference group presented normal colon length and colon tissue, similar to the Control group (supplemental Figure 1(c,d)). These results indicated that treatment with 4-HPR at a dose of 100 mg/kg cannot cause intestinal damage in mice.

### 4-HPR alleviated DSS-induced colonic inflammation in mice

**Figure 2.**
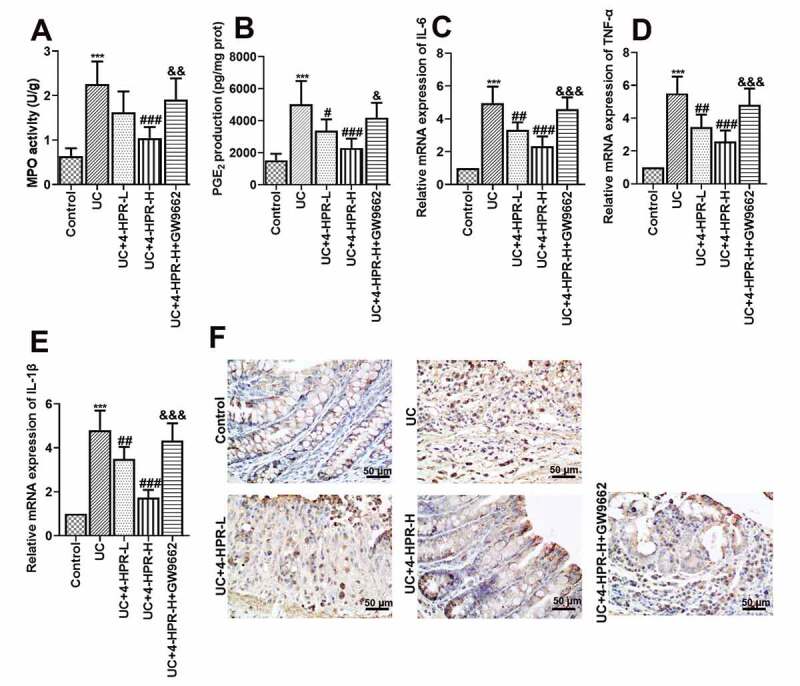
4-HPR alleviated DSS-induced colonic inflammation in mice. (a) MPO activity. (b) PGE_2_ production. (c-e) Relative mRNA expression of IL-6, TNF-α and IL-1β. (f) The expression of COX-2 was examined by IHC at 400× magnification. Data are shown as mean ± SD. ***p < 0.001 vs. Control group; ^#^p < 0.05, ^##^p < 0.01, ^###^p < 0.001 vs. UC group; ^&^p < 0.05, ^&&^p < 0.01, ^&&&^p < 0.001 vs. UC+4-HPR-H group

MPO activity, a biochemical maker for neutrophil infiltration, was detected to assess inflammatory cell infiltration in colonic tissues of mice. PGE_2_, a product of the cyclooxygenation of arachidonic acid released from membrane was associated with colonic inflammation. The results showed that 4-HPR (especially 4-HPR-H) intervention could significantly inhibit the increase in MPO activity and PGE_2_ production induced by DSS, while GW9662 diminished these effects of 4-HPR-H (Figure 2(a,b)). Moreover, increased mRNA expression of pro-inflammatory mediators including IL-6, IL-1β and TNF-α was observed in UC mice, while 4-HPR markedly decreased mRNA levels of these mediators (Figure 2(c-e)). As shown in the results of immunohistochemistry assay, the COX-2 expression was increased in colonic tissue from mice with DSS-induced colitis, however 4-HPR administration reversed this phenomenon (Figure 2(f)). In addition, GW9662 reversed 4-HPR-mediated inhibition of inflammation (Figure 2(c-f)).

### 4-HPR regulated macrophages polarization in colonic tissue from UC mice

**Figure 3.**
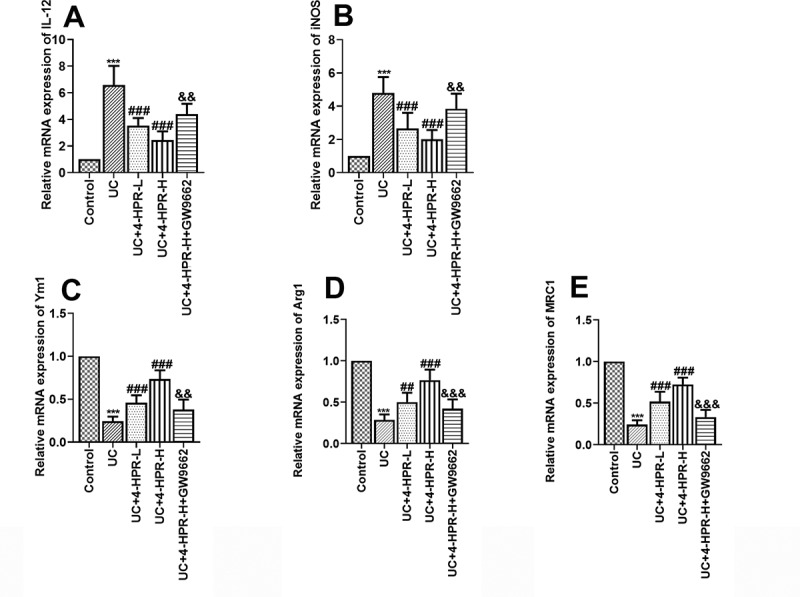
4-HPR regulated macrophages polarization in colonic tissues of UC mice. (a-b) Relative mRNA expression of M1 markers. (c-e) Relative mRNA expression of M2 markers. Data are shown as mean ± SD. ***p < 0.001 vs. Control group; ^##^p < 0.01, ^###^p < 0.001 vs. UC group; ^&&^p < 0.01, ^&&&^p < 0.001 vs. UC+4-HPR-H group

Macrophages polarization plays a critical role in the inflammation response of UC. To explore the anti-UC mechanisms of 4-HPR, we next assessed macrophage polarization in colonic tissues by detecting the mRNA expression of M1 and M2 macrophage markers. Results showed that DSS induction significantly increased the mRNA levels of M1 markers IL-12 and iNOS, while 4-HPR administration inhibited these M1 makers expression. When combined with GW9662, the inhibitory effects of 4-HPR-H on M1 makers disappeared partly (Figure 3(a,b)). Regarding M2 polarization of macrophages in colonic tissues, we detected the mRNA expression of M2 markers including Ym1, Arg1 and MRC1. Results showed that 4-HPR reversed the inhibition of mRNA expression of M2 markers induced by DSS. In addition, GW9662 diminished the positive effects of 4-HPR-H on the expression of M2 markers (Figure 3(c-e)).

### *4-HPR activated PPAR-γ* in vivo *and* in vitro

**Figure 4.**
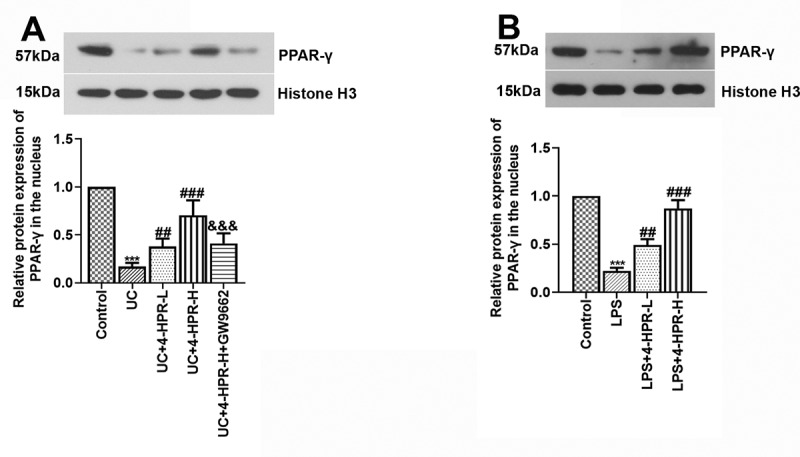
4-HPR activated PPAR-γ in colonic tissues from UC mice and LPS-induced RAW264.7 cells. (a) The nuclear protein expression levels of PPAR-γ in colonic tissues. (b) The nuclear protein expression levels of PPAR-γ in RAW264.7 cells. Data are shown as mean ± SD. ***p < 0.001 vs. Control group; ^##^p < 0.01, ^###^p < 0.001 vs. UC or LPS group; ^&&&^p < 0.001 vs. UC+4-HPR-H group

It has been reported that PPAR-γ pathway is a critical pathway for regulating macrophage polarization and inflammation. In the next experiment, we observed that 4-HPR significantly increased nuclear protein level of PPAR-γ while PPAR-γ inhibitor GW9662 significantly inhibited this increase in colonic tissue from UC mice (Figure 4(a)). 4-HPR might regulating macrophage polarization against inflammation in UC through activating PPAR-γ.

To further explore the mechanism of PPAR-γ relieving UC, a macrophage inflammation model in RAW264.7 cells was established. We firstly measured the protein level of PPAR-γ in the nucleus of RAW264.7 cells. After LPS stimulation, the nuclear protein level of PPAR-γ was dramatically reduced. However, its level was obviously increased by co-treatment with 4-HPR (Figure 4(b)).

### *4-HPR alleviated inflammation and regulated macrophages polarization through activating PPAR-γ* in vitro

**Figure 5.**
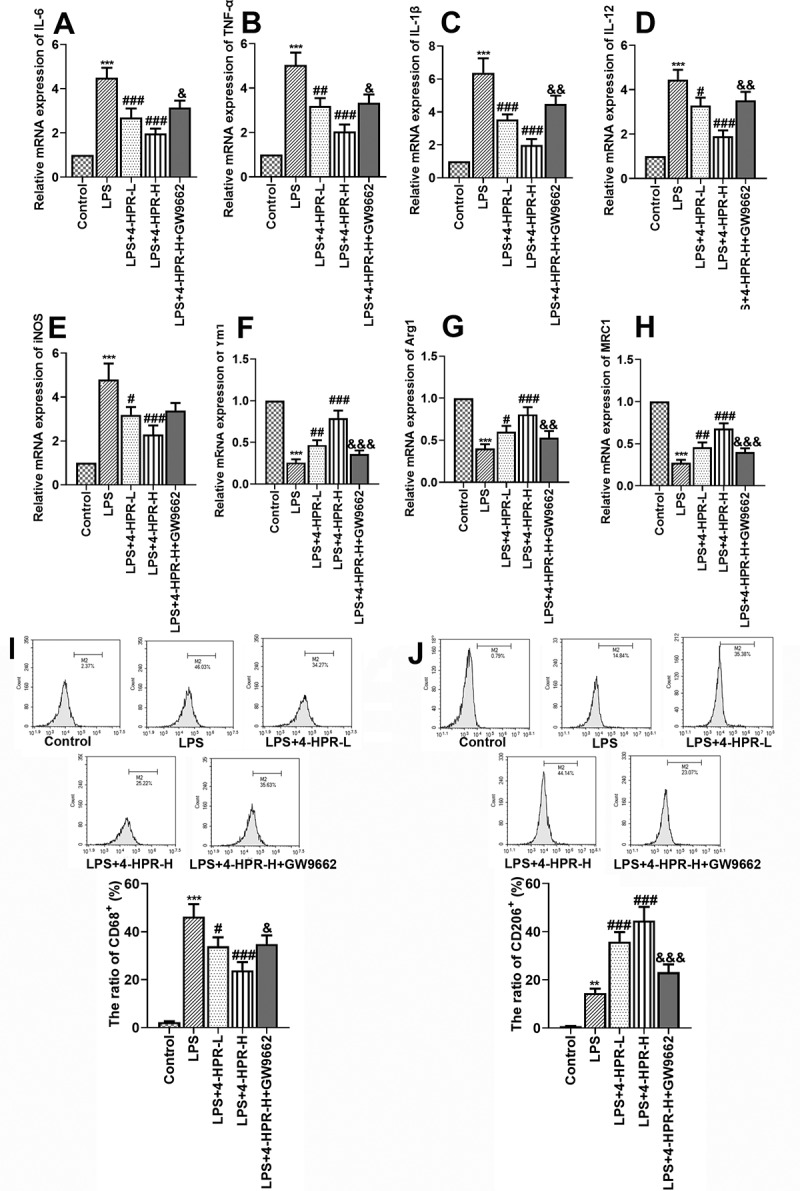
4-HPR alleviated inflammation and regulated macrophages polarization through activating PPAR-γ in LPS-induced RAW264.7 cells. (a-c) Relative mRNA expression of pro-inflammatory cytokines. (d-e) Relative mRNA expression of M1 markers. (f-h) Relative mRNA expression of M2 markers. (i) CD86 (M1 maker)-labeled macrophages were determined by flow cytometry assay. (j) CD206 (M2 maker)-labeled macrophages were determined by flow cytometry assay. Data are shown as mean ± SD. ***p < 0.001 vs. Control group; ^#^p < 0.05, ^##^p < 0.01, ^###^p < 0.001 vs. LPS group; ^&^p < 0.05, ^&&^p < 0.01, ^&&&^p < 0.001 vs. LPS+4-HPR-H group

To confirm that the effects of 4-HPR on UC was related to PPAR-γ, macrophages were pre-incubated with PPAR-γ antagonist GW9662 before LPS and 4-HPR-H incubation. As shown in Figure 5(a-c), the mRNA expression of TNF-α, IL-1β and IL-6 induced by LPS was significantly increased, while treatment with 4-HPR attenuated inflammatory response by decreasing the expression of these factors. Moreover, blockade of PPAR-γ by GW9662 partially reversed the effects of 4-HPR -H on the LPS-induced increase in the expression of pro-inflammatory cytokines.

Next, we explored whether 4-HPR regulated macrophage polarization via activating PPAR-γ *in vitro*. As shown in Figure 5(d-e), 4-HPR effectively downregulated M1 markers expression at mRNA level in LPS- stimulated RAW264.7 cells, however GW9662 abolished the function of 4-HPR on RAW264.7 cells. By contrast, 4-HPR effectively relieved the LPS-induced inhibition of macrophages M2 polarization, while GW9662 partly diminished the expression of M2 markers Ym1, Arg1, and MRC1 by 4-HPR-H. In addition, flow cytometry assay was also performed to identify the M1 and M2 type macrophages after LPS and 4-HPR treatment. Results showed that 4-HPR obviously reduced CD86 (M1 maker)-labeled macrophages and increased CD206 (M2 maker)-labeled macrophages in LPS stimulated conditions. GW9662 abrogated the effects of 4-HPR-H on macrophage polarization by flow cytometry analysis (Figure 5(i,j)).

## Discussion

Synthetic retinoids are a new class of drugs that can effectively alleviate a variety of inflammatory diseases [16,17]. The present study demonstrated that a synthetic retinoid 4-HPR regulated macrophage polarization to protect against UC via the activation of PPARγ. This was supported by several lines of evidence: (1) 4-HPR alleviated DSS-induced UC symptoms and colonic inflammation in mice. (2) 4-HPR reduced the expression of M1 macrophage markers and enhanced the expression of M2 markers in UC mice. (3) 4-HPR activated PPAR-γ in UC mice and LPS-induced RAW264.7 cells. (4) 4-HPR reduced inflammation response, inhibited M1 macrophage polarization and enhanced M2 macrophage polarization in LPS-induced RAW264.7 cells. (5) PPAR-γ antagonist GW9662 effectively eliminated the function of 4-HPR on macrophages polarization both *in vivo* and *in vitro*.

UC is an idiopathic inflammatory disease, which starts in the rectum and usually extends proximally to part of, or even the entire colon [18]. In the present study, although not statistically significant, 4-HPR at a dosage of 50 mg/kg treatment showed slight improvement in the changes of body weight, DAI scores, and colonic histopathology in mice. The results showed that the intraperitoneal administration of 4-HPR at a dosage of 100 mg/kg significantly alleviated DSS-induced colitis evaluated by the DAI and the weight loss assessment, as well as histopathological analysis, indicating the powerful ability of 4-HPR against UC. The pro-inflammatory factors such as IL-6, TNF-α and IL-1β contribute to the pathogenesis of UC [19]. They are key mediators of cellular interactions in the intestine and mediate the control of intestinal homeostasis [20]. In our study, 4-HPR treatment significantly decreased the mRNA expression of these pro-inflammatory factors in the colonic tissue from DSS-induced UC mice and in LPS-stimulated macrophages. The inhibitory functions of 4-HPR on inflammatory response in UC was consistent with previous study which focused on the other model [21]. Furthermore, 4-HPR obviously reduced the level of inflammatory cell infiltration maker MPO, principal inflammation mediator PEG_2_ and its processing enzyme COX-2 in colonic tissues. COX-2 and inflammatory cytokines partly act by recruiting immune cells such as macrophages to infected or damaged tissues. The recruited cells in the infected or damaged tissues participate to the defense response, however, their excess induces tissue damage exacerbating the disease [22]. Results in the present study indicated that the protective effects of 4-HPR against DSS-induced UC was at least partly due to its suppression on the expression of inflammatory factors.

Given the important role of macrophages in the pathogenesis of UC, there is a critical need to understand whether 4-HPR alleviate inflammatory response through regulating macrophage polarization. Results showed that 4-HPR suppressed the mRNA expression of M1 macrophages markers, while upregulated the levels of M2 macrophages markers in colonic tissues of UC mice. It can be accordingly speculated that 4-HPR might regulate macrophage polarization to anti-UC. Stimulated by different signals, macrophages can switch from M1-state to M2-state and vice versa [23]. Specifically, LPS and Th1 cytokines could activate macrophages into an M1 inflammatory state, while Th2 cytokines such as IL-13 could activate macrophages into an M2 phenotype with anti-inflammatory functions [24]. To confirm our speculation, LPS-stimulated RAW 264.7 cells was treated with 4-HPR at different concentrations. Consistent with *in vivo* results, 4-HPR inhibited M1 polarization and enhanced M2 polarization of Raw 264.7 cells. It has been reported that Raw 264.7 cells are identified as ‘innate’ macrophages which can differentiate into M1 or M2 phenotype [25]. Furthermore, Lin et al. has indicated that 4-HPR inhibits LPS-induced iNOS secretion and inflammation [13]. Combining with previous studies and our results, 4-HPR exerted its protective effects on UC by regulating macrophage polarization.

PPAR-γ, a ligand-modulated transcription factor, regulates the expression of genes participating in various processes such as inflammation and redox balance. PPAR-γ, normally residing in the cell cytoplasm, translocates to the nucleus after binding to activating molecules, thus regulating genes expression. Satisfactory anti-inflammatory effects of PPARγ in colitis models has been well investigated [26,27]. Targeted disruption of PPAR-γ in macrophages exacerbated DSS-induced experimental colitis in mice [28]. In addition, PPAR-γ has been reported to be widely expressed in macrophages and PPAR-γ activation skews human monocytes toward to an M2 phenotype [29]. Therefore, we detected the nuclear protein expression of PPAR-γ. We found that 4-HPR upregulated PPAR-γ expression in nuclear from colonic tissue of UC mice and LPS-induced macrophages, indicating the activation of PPAR-γ by 4-HPR. Our data *in vitro* were in accordance with a previous study [13]. Moreover, administration with the specific inhibitor of PPAR-γ GW9662 abolished the inhibitory effects and macrophage polarization regulation of 4-HPR on UC mice and LPS-induced macrophages. Thus, the observed effects of 4-HPR on UC should be at least partly mediated by PPAR-γ. In addition to anti-inflammatory function, 4-HPR also could improve Nrf2 expression and promote Nrf2-antioxidant responsive element (ARE) transcription activity to exert its antioxidant function [30]. The strong relationship has been identified between oxidative stress and the progression of local inflammatory responses in UC [31]. Thus, 4-HPR might not only play an anti-inflammatory function in UC. In the future, we will explore the pharmacodynamics and kinetics of 4-HPR to help understanding of the therapeutic actions and the pharmacological modes of 4-HPR in the treatment of UC.

## Conclusion

The current results demonstrated that 4-HPR alleviated inflammation in DSS-induced UC mice and in LPS-induced RAW264.7 cells through regulating macrophage polarization. According to the *in vivo* and *in vitro* studies, 4-HPR activated PPAR-γ, while PPAR-γ antagonist GW9662 effectively eliminated the function of 4-HPR on macrophages polarization. Hence, 4-HPR could ameliorate experimental UC by modulating macrophage polarization via activating PPAR-γ and 4-HPR might be a potent anti-UC agent.

## Supplementary Material

Supplemental Material

## Highlights

1. 4-HPR alleviated DSS-induced UC symptoms and colonic inflammation in mice.

2. 4-HPR could inhibit M1 and enhance M2 macrophage polarization.

3. PPAR-γ antagonist GW9662 eliminated the function of 4-HPR in UC.
