# Supplementary material for: Fenretinide regulates macrophage polarization to protect against experimental colitis induced by dextran sulfate sodium
Source: Bioengineered. 2020 Dec 31;12(1):151–61. doi: 10.1080/21655979.2020.1859259 (PMC8806340; doi:10.1080/21655979.2020.1859259)
Supplement: Supplemental Material [file KBIE_A_1859259_SM4443.docx]

**Supplemental Figure 1**


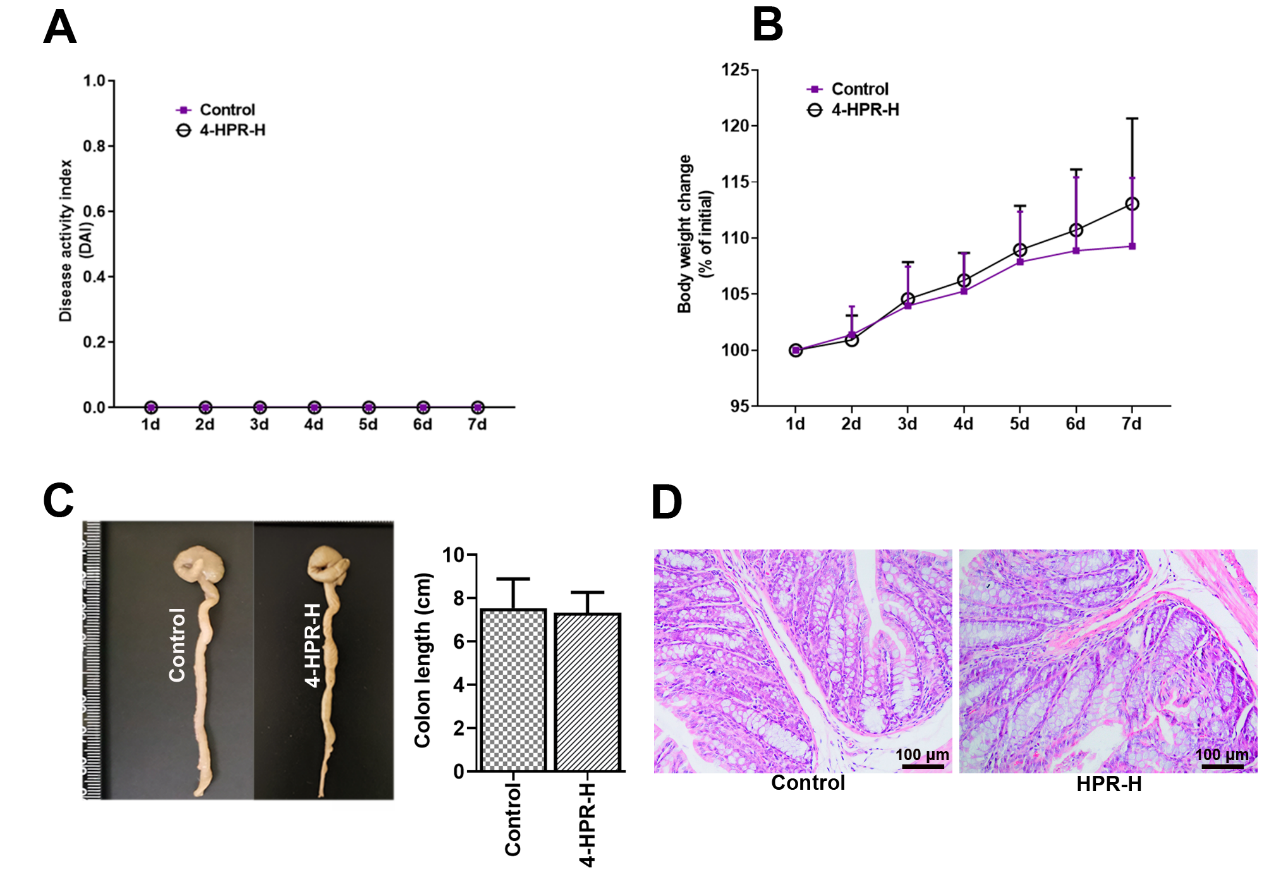


**Figure legends**

**Supplemental Figure 1.** The sole effect of 4-HPR (100 mg/kg) on mice. (A) DAI during experiment period. (B) Body weight change during experiment period. (C) Representative photos of colons and colon length (cm). (D) Representative images of colon tissues with H&E staining (× 200). Data are shown as mean ± SD.
